# Antibacterial and In Vivo Studies of a Green, One-Pot Preparation of Copper/Zinc Oxide Nanoparticle-Coated Bandages

**DOI:** 10.3390/membranes11070462

**Published:** 2021-06-22

**Authors:** Archana R. Deokar, Ilana Perelshtein, Melissa Saibene, Nina Perkas, Paride Mantecca, Yeshayahu Nitzan, Aharon Gedanken

**Affiliations:** 1 Department of Chemistry, Bar-Ilan University, Ramat-Gan 52900, Israel; archanadeokar84@gmail.com (A.R.D.); ch362@mail.biu.ac.il (I.P.); perkasn@mail.biu.ac.il (N.P.); 2 Department of Earth and Environmental Sciences, Particulate Matter and Health Risk (POLARIS) Research Centre, University of Milano, Bicocca, 20126 Milano, Italy; m.saibene2@campus.unimib.it; 3 The Mina and Everard Goodman Faculty of Life Sciences, Bar-Ilan University, Ramat-Gan 52900, Israel; nitzay@biu.013.net.il

**Keywords:** ultrasound irradiation, water, ignition problem, CuO/ZnO NPs, antibacterial, in vivo

## Abstract

Simultaneous water and ethanol-based synthesis and coating of copper and zinc oxide (CuO/ZnO) nanoparticles (NPs) on bandages was carried out by ultrasound irradiation. High resolution-transmission electron microscopy demonstrated the effects of the solvent on the particle size and shape of metal oxide NPs. An antibacterial activity study of metal-oxide-coated bandages was carried out against *Staphylococcus aureus* (Gram-positive) and *Escherichia coli* (Gram-negative). CuO NP-coated bandages made from both water and ethanol demonstrated complete killing of *S. aureus* and *E. coli* bacteria within 30 min., whereas ZnO NP-coated bandages demonstrated five-log reductions in viability for both kinds of bacteria after 60 min of interaction. Further, the antibacterial mechanism of CuO/ZnO NP-coated bandages is proposed here based on electron spin resonance studies. Nanotoxicology investigations were conducted via in vivo examinations of the effect of the metal-oxide bandages on frog embryos (teratogenesis assay—*Xenopus*). The results show that water-based coatings resulted in lesser impacts on embryo development than the ethanol-based ones. These bandages should therefore be considered safer than the ethanol-based ones. The comparison between the toxicity of the metal oxide NPs prepared in water and ethanol is of great importance, because water will replace ethanol for bulk scale synthesis of metal oxide NPs in commercial companies to avoid further ignition problems. The novelty and importance of this manuscript is avoiding the ethanol in the typical water:ethanol mixture as the solvent for the preparation of metal oxide NPs. Ethanol is ignitable, and commercial companies are trying the evade its use. This is especially important these days, as the face mask produced by sonochemistry (SONOMASK) is being sold all over the world by SONOVIA, and it is coated with ZnO.

## 1. Introduction

The increasing resistance of bacteria towards conventional antibiotics strongly motivates designing effective antimicrobial agents to control mortality rates and hospital care costs [1,2]. For instance, methicillin-resistant *Staphylococcus aureus* (MRSA) alone caused 94,360 invasive infections occurred in the United States in 2005; these infections were associated with death in 18,650 cases [3]. Despite it being a nanotechnology era when it comes to conventional antibiotics, one can instead design robust antimicrobial materials to deal with the current scenario of antibacterial resistance. Hence, we consider it an urgent need to design a robust antimicrobial material to deal with the current scenario of antibacterial resistance. Based on nanotechnology, during the last few years several research groups put forth efforts to deal with the aforementioned antibacterial resistance [4,5,6,7]. Rotello and coworkers highlighted the multiple nanoparticle-based approaches to eliminate bacterial infections, providing crucial insights into the design of elements that play critical roles in creating antimicrobial nanotherapeutics. They focused on the pivotal role played by NP-surface functionality in designing nanomaterials as self-therapeutic agents and delivery vehicles for antimicrobial cargo [8].

The antibacterial properties of organic and inorganic nanoparticles (NPs), and carbon based nanomaterials, including graphene, have been explored [9,10,11,12,13,14,15,16]. Among them, silver and semiconductor NPs, such as titanium dioxide and zinc oxide NPs, have been explored thoroughly [17,18,19]. Few NPs have been commercialized though, as toxicity issue of these NPs remains an unresolved issue [20]. The toxicity of NPs might arise as a result of the use of toxic chemicals during the synthesis processes. Additionally, the use of volatile organic solvents during bulk scale synthesis of NPs might lead to higher chances of accidental fires due to harsh experimental conditions, such as high temperatures. Practising green and sustainable chemistry for the synthesis of NPs would be optimal, as it would allow avoiding the use of hazardous chemicals as well [21]. Safer chemicals will lead to less toxicity and less chances of accidents during bulk scale synthesis. In our group, the ultrasound-assisted one-pot synthesis and deposition of magnesium fluoride, yttrium fluoride, chitosan, copper oxide/zinc oxide (CuO/ZnO) NPs on cotton, catheters, polymer, glass, and teeth has been explored to study their antibacterial/antibiofilm properties [22,23,24,25,26,27,28]. Ultasound-assisted coating is green, efficient and cost effective, as it consumes less energy, solvent, and materials. Further coating is possible without the addition of any binder [29]. In sonochemistry, chemical effects of ultrasound arise from “acoustic cavitation,” meaning continuous formation, growth, and implosive collapse of bubbles in the liquid, which creates an unusual chemical environment. “Cavitation” depends on several factors, such as the viscosity of the solvent and the ultrasonic intensity [22]. Physical and chemical properties of NPs can be enhanced with different morphologies of NPs by varying several of the aforementioned parameters, e.g., the solvent. Ali et al. studied the formation of nano/microparticles of vermiculite by varying different sonication conditions, such as the solvent, the reaction time, and the temperature [30].

In our previous study [31], we carried out ultrasound-assisted coating of metal oxide NPs onto cotton, which has been proven to be effective against multi-drug resitant bacterial strains. A solvent ratio of 90:10 ethanol:water (v:v) was employed for coating these NPs onto cotton bandages. This was performed in the course of an EC project called SONO in FP7. The commercial companies complained about the use of ethanol, which is an ignitable material and induces extra cost for its removal. Herein, we report the first successful coating of copper and zinc oxide NPs onto cotton using the universal solvent, water. This method will be beneficial economically and environmentally, since water is non-toxic and non-flammable. Water-based CuO NP-coated bandages were equally as effective as ethanol-based CuO NP-coated bandages for complete eradication of both *S. aureus* (Gram-positive) and *Escherichia coli* (Gram-negative) bacteria.

## 2. Experimental

### 2.1. Deposition/Coating Procedure of CuO/ZnO NPs on Bandages

The coating of NPs on fabric was done using roll to roll sonochemical installation [32]. Copper/zinc acetates (0.01 M) were dissolved in 400 mL of double distilled water (ddH_2_O). A 9/1 ethanol:water volume ratio solution was obtained after adding 3.6 L of ethanol. Please note that whenever we refer to ethanol solution it means ethanol:water 9:1 (v:v). In case of water synthesis, ethanol was replaced by ddH_2_O. The solutions were heated and when a temperature of 60 °C was reached, 7–10 mL of an aqueous solution of ammonium hydroxide (28–30%) was injected into the reaction container to adjust the pH to ~8. At the end of the reaction, the color of the fabric changed from white to brown in the case of CuO NPs, whereas in case of ZnO NPs it remained white due to a curdy white precipitate of ZnO NPs. The coated bandages were cleaned with ddH_2_O and once with ethanol, and then dried under vacuum. Powders resulting from coated bandages were collected, dried, and subjected to further characterization.

### 2.2. Characterization of CuO/ZnO NPs and Bandages 

Powders obtained from roll to roll coatings of the corresponding metal oxide NPs were subjected to XRD (Bruker D8 diffractometer employs Cu K alpha radiation). Morphologies and particle sizes of water/ethanol-based metal oxide NPs were observed by HR-TEM (high resolution transmission electron microscopy) (JEOL 2100, with accelerating voltage of 200 kV). Coated metal oxide weight percentages were analyzed by ICP (ion coupled plasma—ULTIMA 2). Dried metal-oxide-coated bandages were coated with conducting carbon and subjected to ESEM (FEI QUANTA 200F device) to confirm the uniform distribution of NPs on bandages. BET (Brunauer-Emmett-Teller) measurements were carried out as well.

### 2.3. ESR Measurement

ESR (electron spin resonance) coupled with a spin trap of 5,5-dimethyl-1-pyrroline-N-oxide (DMPO, Sigma, St. Louis, MO, USA) was employed to determine the reactive oxygen species (ROS) production abilities of water/ethanol-based CuO/ZnO NP-coated bandages and the corresponding powders as well. Typically, for bandages, a 1.5 × 1.5 cm^2^ piece of metal oxide NP-coated bandage was immersed in 180 µL of double distilled water (ddH_2_O). Further, 20 µL of DMPO was allowed to react with the bandages for 10 min, and subsequently, 90 µL of solution was withdrawn by a syringe into a gas-permeable Teflon capillary (Zeus Ind., Raritan, NJ, USA) with an inner diameter of 0.082 cm, 0.038 inch wall thickness, and 15 cm length. Each capillary was folded twice, inserted into a narrow quartz tube open at both ends, and then placed in the ESR cavity. The ESR measurement conditions were as follows: 9.74 GHz frequency; microwave power, 20 mW; scan width, 65 G; resolution, 1024; receiver gain, 2 × 10^5^; conversion time, 82 ms; time constant, 655 ms; sweep time, 84 s; scans, 2; modulation frequency, 100 kHz. After acquisition of the data, the spectra were processed using Bruker WIN-EPR version 2.11 for baseline correction. The peak intensity, which is proportional to the amount of ROS, was calculated by double integration of the peak signals, and the intensity is expressed in arbitrary units.

A simulation of the recorded spectra was performed using an algorithm provided in WINSIM proGram, which is available from the NIEHS (National Institute of Environmental Health Sciences) at (http://epr.niehs.nih.gov/pestmans/winsim.html).

### 2.4. Colony Forming Unit Method (CFU) 

The antibacterial studies of ZnO/CuO NP-coated bandages were evaluated by using the CFU method against *S. aureus* and *E. coli* bacterial strains. Overnight-cultured bacterial strains were transferred into a fresh Tryptic Soy Broth (TSB) medium and agitated for 4 h at 37 °C; then, 10^8^ cell cultures were harvested by centrifugation and washed with fresh 0.9% sodium chloride (NaCl) solution. ZnO/CuO coated bandages of 1.5 × 1.5 cm were allowed to react with 2 mL of one bacterial strain for 60 min. Aliquots of 200 µL were taken out at different time intervals (0, 10, 30 min) and serially diluted with a fresh 0.9% NaCl solution, and further plated onto agar for quantitation. 

### 2.5. In Vivo Toxicity Assay 

The toxicity of the differently coated bandages was assessed by the standard frog embryo teratogenesis assay using *Xenopus* (FETAX, ASTM, 1998), which was modified to allow embryos to stay in direct contact with the bandages. Embryos were incubated in contact with uncoated and coated bandages for 96 h, from the blastula to the larval stage. The protocol used for this modified FETAX is briefly reported below. Adult *Xenopus laevis* were maintained in aquaria with dechlorinated tap water at a 22 ± 2 °C, in an alternating 12 h light/dark cycle, and were fed a semi-synthetic diet three times a week. The mating behavior of adult males and females was stimulated by Human Chorionic Gonadotropin (HCG) injection. After natural breeding, normally cleaved embryos at the blastula stage (stage 8), 5 h post-fertilization (hpf), were selected for testing, and after dejellying in 2.25% L-cysteine, 25 blastulae were placed in 8.0 cm glass petri dishes containing 30 mL of FETAX solution. On the bottom of each petri dish, the differently coated bandages to be tested were fixed, after being soaked for 15 min in Milli-Q water. All petri dishes were incubated in a thermostatic chamber at 23 ± 0.5 °C until the end of the test (96 hpf) in static conditions; each day the petri dishes were observed and dead embryos were removed. Mortality and malformation data were generated as endpoints of the assay. For each experimental group, the number of dead larvae was recorded; survivors were anaesthetized with 100 mg/L MS-222 and evaluated for single malformations under a dissection microscope. At the ends of the bioassays, surviving normal larvae were formalin fixed for growth retardation measurements. The number of malformed larvae was recorded, and body length (head to tail) was measured to derive the growth retardation effect. The assay was repeated at least three times under the same experimental conditions. The numbers of dead embryos versus their total numbers at the beginning of the tests led to the mortality percentages; and the numbers of malformed larvae versus the total numbers of surviving ones gave the malformed larva percentages. The relationship between the control and treated groups along with the percentages of dead and malformed larvae were investigated with Chi-square tests. To evaluate differences in growth retardation among groups, ANOVA was used. Statistical comparisons were considered to be significant at the 95% level (*p* < 0.05).

## 3. Results and Discussion

One of the motivations for the current study was the evaluation of the potential toxicity of metal oxide NPs of various shapes. From our observations, the solvent is one of the parameters that influences the shape and size of the sonochemically-produced metal oxide NPs. Therefore, the synthesis was carried out in a primarily ethanol solution or in water. The products obtained in water and ethanol-based synthesis were characterized by various techniques and compared; the results are presented below.

### 3.1. Chemical Composition and Morphology 

The coatings of CuO/ZnO NPs on cotton bandages were analyzed by ICP analysis. It is worth mentioning that bandages coated with metal oxide NPs using water as a solvent with equal molarities demonstrated larger amounts of coated material compared with ethanol equivalents. Equal amounts of metal oxide on bandages are essential for comparing bacterial killing efficiency. We found that water-based metal oxide NPs with initial precursor concentrations of 0.0050 M (zinc acetate) and 0.0040 M (copper acetate) demonstrated approximately equal weight percentages of metal oxide NP coatings corresponding to ethanol-based synthesis of metal oxide NPs with the initial precursor concentration of 0.01 M (Table 1).

**Figure 1:**
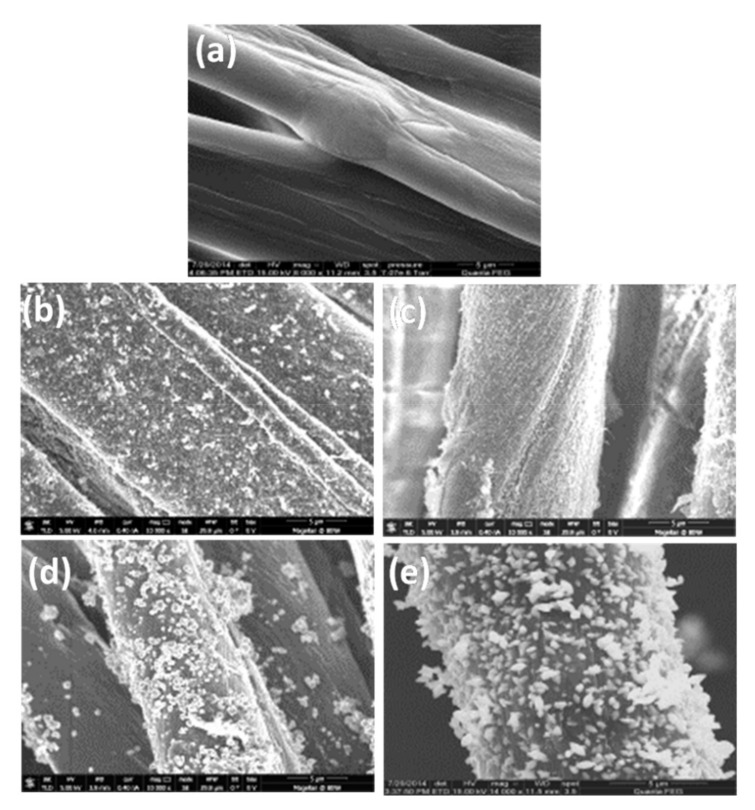
ESEM images of (**a**) pristine cotton, cotton fabric coated with CuO and ZnO NPs in ethanol (**b**,**d**) or in water (**c**,**e**); scale bar is 5 µm.

CuO/ZnO NP-coated bandages in water/ethanol were subjected to ESEM (Figure 1) to observe their morphologies, and they were compared with pristine bandages. Both water/ethanol synthesized CuO/ZnO NP-coated bandages (Figure 1b–e) demonstrated homogenous and dense coatings of metal oxides as compared to the smooth pristine bandages (Figure 1a–e). 

**Figure 2:**
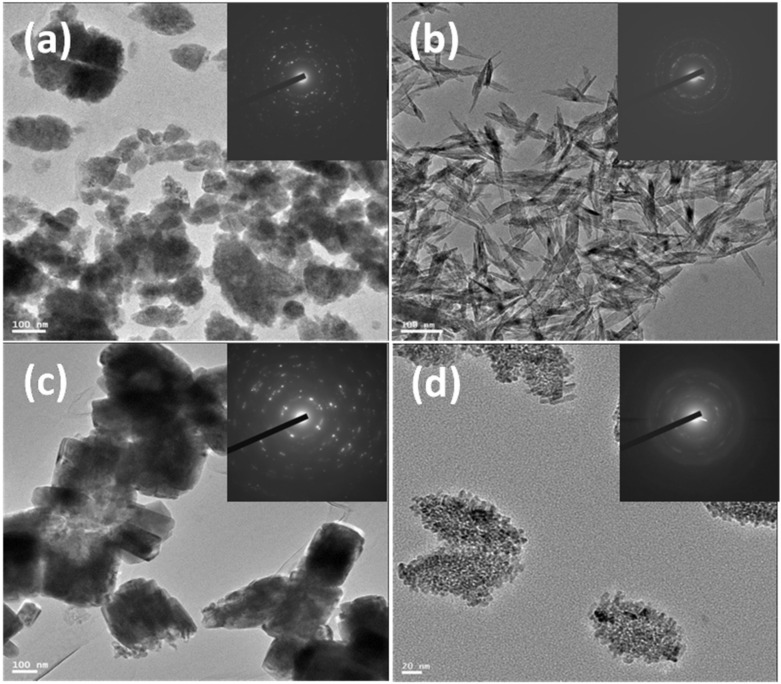
HR-TEM images of water-synthesized ZnO and CuO NPs (**a**,**b**); scale bar 100 nm; (**c**,**d**) represent images of ethanol-based synthesized ZnO and CuO NPs, respectively; scale bars 100 and 20 nm.

The ZnO and CuO NPs synthesized with water differ in shape and size compared with the products of ethanol-based synthesis. A detailed analysis of shapes and sizes of metal oxide NPs was carried out by HR-TEM. As expected, solvents played the major role in controlling the shapes and sizes of the metal oxide NPs. Figure 2 presents the HR-TEM images of ZnO and CuO NPs synthesized in water or in ethanol. The inset indicates SAED (selected area electron diffraction) of the NPs. All the synthesized NPs were crystalline, and a typical ring diffraction pattern was observed. The diffractions of each metal oxide match very well with the XRD result presented in Figure 2. Water-based ZnO NPs (Figure 2a) demonstrated a rugby ball shape with an average length of ~109 nm and a width of ~70 nm (aspect ratio ≈ 1.5). Ethanol-based ZnO NPs (Figure 2c) were rectangular in shape, and the average length and width were ~270 and ~166 nm, respectively; they had an aspect ratio of ~1.6. Leaf-like structure was observed for CuO NPs synthesized in water, and the average length and width were ~113 and ~25 nm, respectively; the aspect ratio was ~4.52 (Figure 2b). Aggregates of ~130 nm were observed among ethanol-based CuO NPs, and the individual particle size was ~7 nm (Figure 2d). CuO NPs synthesized in ethanol demonstrated a much smaller particle size compared with water-synthesized particles. It is clearly seen that the type of solvent during the hydrolysis of metal acetates has impacts on the shape and the size of the NPs. According to the BET method, the average surface areas for CuO NPs synthesized in ethanol and water were 76 and 51 m^2^/g, respectively, whereas in case of ZnO NPs synthesized in ethanol and water, both average surface areas were 10 ± 1 m^2^/g. These similar values were due to the resemblance in size of the ZnO NPs fabricated by these solvents. BET results were in good agreement with HR-TEM results.

**Figure 3:**
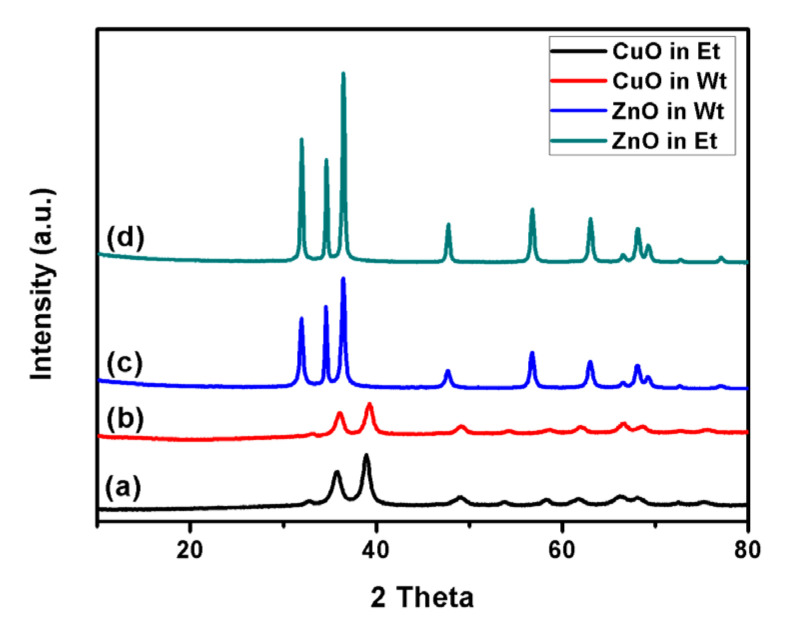
XRD spectra of CuO NPs synthesized in ethanol (**a**) or in water (**b**). ZnO NPs synthesized in water (**c**) or in ethanol (**d**).

Figure 3 represents the XRD patterns of CuO and ZnO NPs synthesized in water and ethanol. The crystalline structure of neither zinc oxide nor copper oxide was influenced by the solvent used during the hydrolysis process. Identical reflection peaks were obtained in both solvents, and the peaks well match the patterns identified in the previous studies (PDF files 89-7102 and 80-1916) [29]. Moreover, even the intensities of the reflection peaks were similar for both solvents. 

### 3.2. ESR Studies

**Figure 4:**
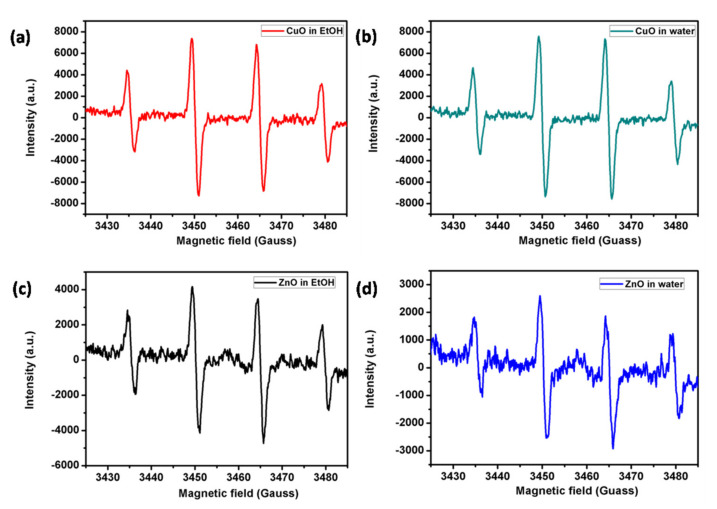
ROS generation by CuO and ZnO NP-coated bandages synthesized in ethanol (**a**,**c**) or water (**b**,**d**) in the presence of DMPO alone.

ROS production of NPs was determined by the ESR technique, while employing DMPO as a spin trap. Four resolved peaks generated from the DMPO-OH adduct identified the presence of hydroxyl radicals in the metal-oxide NP-coated bandages (Figure 4). 

CuO NP-coated bandages (Figure 4a,b) made in water or ethanol gave rise to more intense peaks compared to ZnO NP-coated bandages made in either water or ethanol (Figure 4); this was due to CuO NPs being smaller particles compared to ZnO NPs (Figure 2). Smaller particles possess greater surface area to volume ratios, and hence induce more ROS production. ESR studies were in good accordance with BET and antibacterial studies. ZnO NPs synthesized in water or ethanol demonstrated weak ESR signals, indicating a poor ability to generate hydroxyl radicals.

### 3.3. Antibacterial Studies

**Figure 5:**
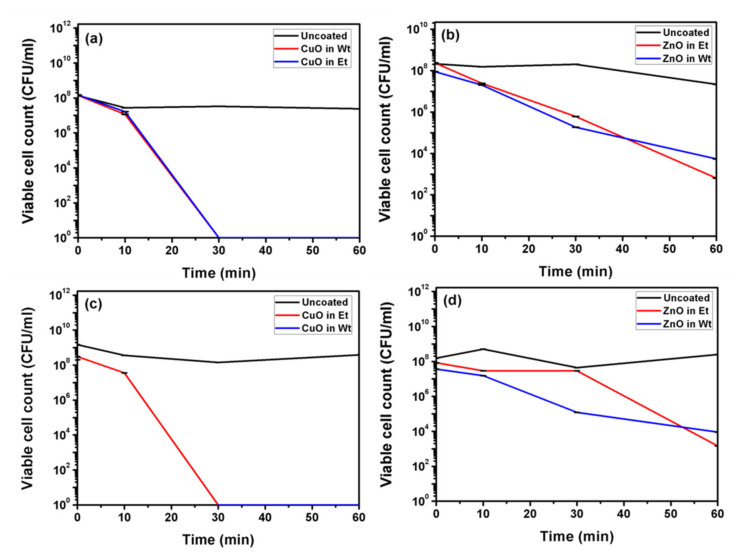
Antibacterial studies of CuO and ZnO NP-coated bandages in water or ethanol against *S. aureus* (Gram-positive) (**a**,**b**) and *E. coli* (Gram-negative) (**c**,**d**).

Figure 5 represents antibacterial activity for water and ethanol-based synthesized ZnO and CuO NP-coated bandages. In the case of CuO NP-coated bandages, considering both water and ethanol, complete eradication of both *S. aureus* and *E. coli* bacteria was observed within 30 min. (Figure 5a,c), whereas in 60 min, ZnO NP-coated bandages (water or ethanol-based) revealed only 5-log reductions for both bacterial strains (Figure 5b,d). The antibacterial activities of the coated bandages are in accordance with the ESR and BET studies. CuO NPs are smaller; i.e., they have more surface area compared to ZnO NPs, and hence better antibacterial activity. Herein we demonstrate that better ROS production by CuO NPs could lead to better antibacterial activity compared to ZnO NPs.

**Figure 6:**
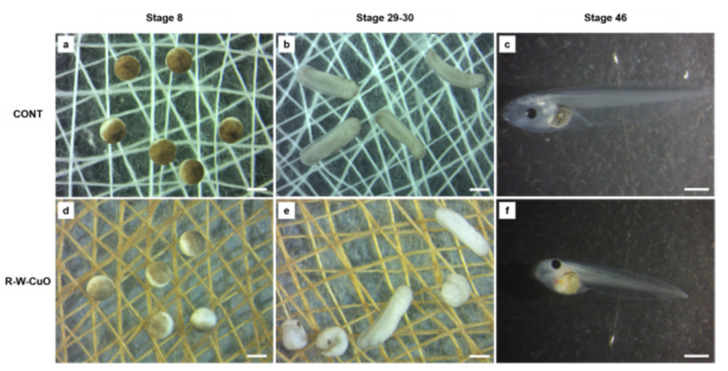
Stereomicroscopic images showing the experimental set up, with embryos in direct contact with uncoated cotton bandages (**a**,**b**) and CuO NP-coated bandages in water (W-CuO) (**d**,**e**), fixed at the bottom of glass petri dishes. Embryos were photographed at the beginning (stage 8) (**a**,**d**) and at the middle (stage 29–30 of the test). Larvae at the end of the test (stage 46) were screened for single malformations: (**c**), control larva; (**f**), W-CuO exposed larva showing irregular gut and body length shortening. Bars = 1 mm.

Commercialization of CuO and ZnO NP-coated bandages requires in vivo or vitro toxicity screening. Hence, we have carried out an in vivo toxicity study of bandages using the FETAX (ASTM, 1998) standard, which we modified to allow embryos to stay in direct contact with the bandages. Embryos (Figure 6, stage 8) were incubated in contact with uncoated and coated bandages for 96 h from the blastula to the larval stage. Very little, non-significant embryo lethality was observed for all the bandages screened, except the ethanol-based CuO NP (E-CuO) coated bandages (Figure 6). Only E-CuO showed a significant difference in lethality (Table 2).

**Figure 7:**
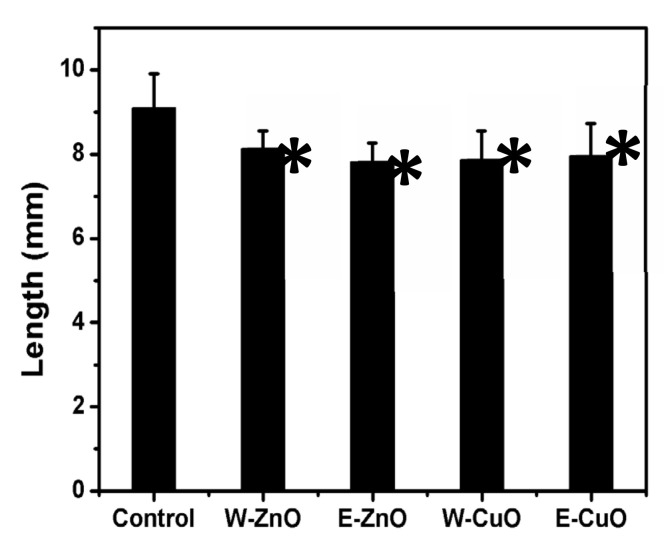
Growth retardation effects in embryos exposed to water or ethanol-based ZnO and CuO NP-coated bandages. * Statistically different from the control (ANOVA, *p* < 0.05).

All bandages significantly increased the malformation percentage in exposed embryos. Regardless, the percentage of malformed embryos for most of the types of coated bandages was lower than 40%, testifying to a weak teratogenic capacity. Indeed, our results were below the EC_50_ (effective concentration for 50% of the embryos used), which is usually combined with the LC_50_ (lethal concentration for the 50% of the embryos used) for calculating the teratogenic index. Only embryos grown in contact with ethanol-based ZnO coated bandages showed a high malformation percentage (71.3%), a value significantly higher than those of the corresponding water-based ZnO or CuO NP-based coatings (Table 2). The embryo growth retardation effects were significant and comparable in all the treatment groups, confirming that all the materials used exert baseline toxicity toward developing *Xenopus* embryos, in line with previous studies (Figure 7) [33].

In conclusion, according to the present results, water-based metal oxide NP-based coatings are less harmful to embryo development; in particular, we did see rescued embryo mortality with the CuO coating and rescued embryo malformation with ZnO bandages, which allows us to consider water-based bandages as safer than ethanol-based ones. Our in vivo studies are in accordance with ESR (Figure 4): ethanol-based metal-oxide-coated NPs gave rise to an intense hydroxyl radical signal compare to water-based metal-oxide-coated NPs. The involvement of ROS has been previously demonstrated in sonochemically synthesized ZnO NP-induced toxicity [34]. This evidence was obtained by exposing *Xenopus* embryos to ZnO and CuO NPs synthesized in ethanol, and even in that case, only ZnO NPs induced embryo malformation. Diffraction patterns (Figure 2) of both water and ethanol-based synthesized ZnO NPs demonstrated a hexagonal crystal structure, so the possibility of adverse effects due to the chemical structure of ZnO NPs can be neglected. Additionally, considering that such adverse effects have been attributed to the direct contact between embryo tissues and NPs, rather than Zn ions’ dissolution [35], it is possible to hypothesize that the higher toxicity induced by ethanol ZnO NPs may be dependent on the peculiar physical structure, which leads to ROS production once in contact with biological tissues. Of course, this aspect, as well as the specific contribution to toxicity of the dissolved ions, merits further experimental efforts. 

## 4. Conclusions

In conclusion, one-pot synthesis of CuO and ZnO NP coatings on fabrics was successfully performed in water for the first time. CuO NP-coated bandages in water or ethanol were found to be more effective against both *S. aureus* and *E. coli* compared to ZnO NP-coated ones. Herein, our study demonstrated that ethanol can be replaced by water for the synthesis of metal-oxide NPs. This could be a great way to avoid ignition accidents during bulk scale synthesis of metal-oxide NPs in commercial companies. Moreover, on the basis of in vivo studies of CuO/ZnO NP-coated bandages against FETAX, we conclude that water-based metal-oxide-coated bandages were less toxic compared to ethanol-based ones. Hence, these coated bandages could bring about a revolution in combating hospital acquired infections, and hence might contribute to a lower mortality rate and lower hospital care costs. 

## Figures and Tables

**Table 1:** Amounts of metal coated on the cotton fabric.

Precursor Concentration (Molar)	Coated Bandages	ZnO%	CuO%
0.01	ZnO in ethanol	0.74	-
0.01	CuO in ethanol	-	1.38
0.0050	ZnO in water	0.71	-
0.0040	CuO in water	-	1.50

**Table 2:** Embryotoxic effects in *X. laevis*.

	Bandages				
	**Control**	**W-ZnO**	**E-ZnO**	**W-CuO**	**E-CuO**
Utilized embryos (n)	151	124	151	122	126
Dead embryos (n)	2	6	1	6	20
Mortality (%)	1.3	4.8	0.7 °	4.9	15.9 ** °°
Living larvae (n)	149	118	150	116	106
Malformed larvae (n)	5	30	107	43	39
Malformed larvae (%)	3.4 **	25.4 **	71.3 ** °	37.1 **	36.8 **

** Chi square test; *p* < 0.001 vs Control, ° Chi square test; *p* < 0.05 water vs. ethanol for the same metal oxide NPs. °° Chi square test; *p* < 0.001 water vs. ethanol for the same metal oxide NPs.
